# Camera-on-tip endoscope for *in vivo* cardiovascular diagnostics and surgical guidance

**DOI:** 10.1364/BOE.543373

**Published:** 2024-12-03

**Authors:** Simon T. Sørensen, Walter Messina, Lorenzo Niemitz, Claire O’Dowling, Piotr Buszman, Stefan Andersson-Engels, Ray Burke

**Affiliations:** 1 Biophotonics@Tyndall, IPIC, Tyndall National Institute, Lee Maltings, Dyke Parade, Cork, Ireland; 2 Centre for Research in Vascular Biology, APC Microbiome Ireland, University College Cork, Cork University Hospital, Cork, Ireland; 3 Center for Cardiovascular Research and Development, American Heart of Poland, Poland; 4 University College Cork, School of Physics, Cork, Ireland

## Abstract

Cardiovascular imaging with camera-on-tip endoscopes has the potential to provide physiologically relevant data on the tissue state and device placement that can improve clinical outcomes. In this work, we review the unmet clinical need for image-based *in vivo* cardiovascular diagnostics and guidance for minimally invasive procedures. We present a 7 Fr camera-on-tip endoscope with fibre-coupled multispectral illumination that includes methods for imaging in a blood-filled field of view (FOV). We demonstrate that the endoscope can be navigated from the femoral artery to cardiac regions such as the left atrium and left ventricle in a porcine model, where *in vivo* images of the cardiac walls are recorded. We further show that physiologically relevant parameters such as heart rate and respiration can be extracted from the images and that changes to tissue state can be inferred from the imaging data. Finally, a methodology for merging the imaging data with diffuse reflection spectroscopy (DRS) recorded through the optical fibre is outlined.

## 1. Introduction

Recent advances in highly sensitive detectors and novel light sources across the visible and near-infrared spectrum have opened up new possibilities in biomedical sensing. Furthermore, CMOS camera technology with a reduced footprint derived from the enormously high volume smartphone market has been repurposed, and such micro-image sensors with sub-millimetre dimensions are now commercially available for endoscopy applications [1]. From a spectroscopy point of view, Diffuse Reflectance Spectroscopy (DRS) has seen extensive use in tissue differentiation research, distinguishing healthy tissue from tumor tissue [2–5], and for assessing the vulnerability of coronary artery plaques [6]. This allows for simple integration requiring two spatially separated optical fibres and is a modality that is integrated into the camera-on-tip endoscope system in this work. It also lends itself to combination with other systems, such as an image sensor, since illumination fibres can be switched to be used as DRS excitation and collection fibres using an optical switch.

Imaging the cardiovascular space has become part of the standard of care for cardiovascular illnesses. The scale of this imaging can range from very large magnetic resonance imaging (MRI) and computed tomography (CT) devices [7,8] to millimetre-sized intravascular imaging in intravascular-ultrasound (IVUS) [9], optical coherence tomography (OCT) [10,11], or scanning fibre endoscope (SFE) systems [12]. Imaging wavelengths and technologies span a similarly broad spectrum from X-ray [13] through the visible to the infrared [14]. Each of these optical and acoustic modalities provide different penetration depth, spatial and spectral resolution. Camera systems and CMOS technology promise to play a greater role as the technology develops further and is adopted more in biomedical research [15–17] and clinical applications [14]. Angioscopy based on fibre optics has already been demonstrated as a potentially powerful adjunctive tool in cardiovascular medicine with applications such as bypass and thromboembolectomy procedures, endovascular neurosurgery procedures, and general peripheral vascular operations [18,19]. Autofluorescence has been demonstrated as an indicator of RF lesions in cardiac tissue captured through imaging fibre [20]. The goal in each case is to provide real-time intravascular image data to a clinician, while minimising the use of harmful fluoroscopy and providing information in situ that would otherwise be impossible to obtain. As imaging technologies develop further, they promise to provide unprecedented information that can drive lifesaving decisions.

Recent work has investigated transthoracic systems based on micro-CMOS imagers. Karimov *et al.* performed a transapical and percutaneous cardiac procedure to visualise intracardiac structures in bovine models with two different cardioscopic systems [21]. Fagogensis *et al.* applied autonomous intracardiac navigation using haptic vision to navigate an imaging catheter to the cardiac walls in animal models [22]. Alotaibi *et al.* used a commercial bronchoscope with a diameter of 5-6 mm to visualise the intracardiac anatomy in an *ex-vivo* porcine heart [23]. Opfermann *et al.* demonstrated a novel videoscope to percutaneously access the pericardial space under direct visualisation in porcine models [24]. Tahmasebi *et al.* presented the CathCam angioscope to image the navigation of a guidewire through atherosclerotic plaque, which was demonstrated on *ex vivo* lesions and *in vivo* in a porcine model [25].

Despite the rapid technological advancements of CMOS technology, challenges remain, especially regarding the integration, to fully meet the clinical needs required for *in vivo* diagnostics and surgical guidance deep within the human body. In this paper, we present a multimodal camera-on-tip endoscope for *in vivo* cardiac applications. Specifically, we are proposing that the image sensor can provide not just a visual aid to the clinician but also quantitative information about the tissue state. As an example of the clinical potential, we demonstrate *in vivo* imaging of the cardiac walls of a beating heart of a porcine model over a duration of several minutes with the camera-on-tip endoscope inserted through the femoral artery. We further show that physiological parameters such as heart and respiration rate can be extracted directly from these images. By occluding the left anterior descending artery, we show that changes to the tissue state can be inferred from the imaging data. Finally, we demonstrate that combining endoscopic imaging with DRS is a promising technique that can provide additional physiologically relevant information about tissue composition and state. It is well known that tissue with different levels of oxygenation, i.e., a different ratio of oxygenated and deoxygenated hemoglobin present, will have a different spectral response [26,27]. In cardiac tissue, monitoring tissue ischemia, that is areas of reduced or restricted blood flow, is of clinical significance. For example, direct imaging of tissue vascularisation can potentially provide clinical validation of circumferential ablation of the left atrial appendage for the treatment of persistent atrial fibrillation.

## 2. Methods

### 2.1. Camera-on-tip endoscope system

The sensor used in this work is the AMS NanEYE2D RGB fitted with a grated index (GRIN) lens with a field of view (FOV) of 120 degrees in air, and F number of 2.8. Due to operation in a liquid field, the effective FOV *in vivo* is reduced to approximately 90 degrees. The GRIN lens system provides a simple and effective general lens system for the micro image sensor, with effective focus from 3.0 mm to infinity and an optimal focus at 8.0 mm. However, a custom lens solution is expected to be able to further improve image quality, especially when the design is application specific.

**Fig. 1.**
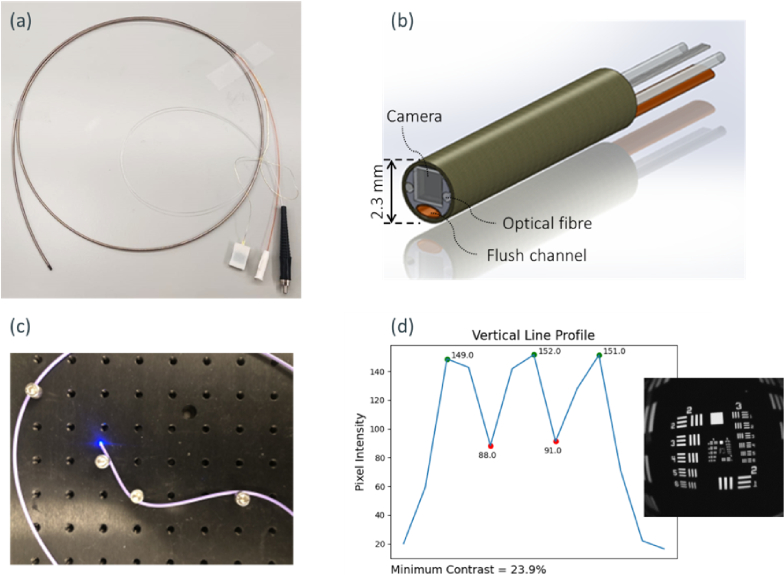
(a) Image of the camera-on-tip endoscope. The proximal end has three connectors for the camera communication, fibre connector and saline flush channel, respectively. (b) CAD drawing showing camera-on-tip sensor at the distal end of the endoscope. The outer diameter is 2.3 mm which includes the 1 ${\text{mm}}^{2}$ imaging sensor, two Ø225 µm illumination fibres and flush channel. The rigid tip length is 5 mm. (c) Mechanical bend test of the endoscope showing light delivery through at radius close to the fibres’ minimum radius of 42 mm. (d) Resolution characterisation with USAF1951 target, showing the vertical line-profile of Group 3-4 at 4 mm distance, corresponding to a resolution of 12.70 lp/mm.

The image sensor is integrated into a 3D printed alignment holder along with two 200 um, 0.39 NA optical fibres (Thorlabs FT200EMT) and a saline flush channel. The flush channel consists of 1.20 mm OD braided polyimide tubing (Nordson medical, USA). The system is manually operated using a syringe which also acts as the saline reservoir. The system sits within a 2.30 mm OD catheter tubing, the dimensional requirement of which was informed by the target cardiovascular application. The endoscope has a short rigid tip length of 5 mm needed for cardiovascular applications. An image of the probe and drawing of the distal module can be seen in Fig. 1. The detailed mechanical design, assembly, and integration of the system is described in [28]. Illumination can be coupled to the two illumination fibres at the proximal end and is delivered using custom-built light sources. Delivering the illumination via fibre rather than from emitters mounted at the distal end near the image sensor, gives the advantage of flexibility in illumination to beyond traditional RGB imaging. Figure 1 further shows examples of mechanical bending and resolution characterisation to demonstrate the endoscope’s flexibility and optical resolution. The characterisation is performed at a 4 mm working distance to mimic the final use case. The observed spherical aberrations are because of the GRIN lens package and will be improved in future work with different lens designs and software corrections.

Due to the lack of low-cost commercial multispectral sources with high customisability, sources are built in house. The endoscope backend system is designed to be portable and compact to accommodate the working environment of the operating theatre. Laser illumination allows for narrow bandwidth illumination targeted to probe specific spectral regions of interest as well as allowing for recombination of the illumination wavelengths, or for sequential or synthetic white-light illumination, to reconstruct colour image [29]. Laser also provides a high coupling efficiency for fibre coupling. The source used primarily in this work is a laser source combining 6 wavelengths from the VIS to NIR. The coherent nature has shown some limitations which will be discussed later.

### 2.2. Multimodal and multispectral biomedical imaging

**Fig. 2.**
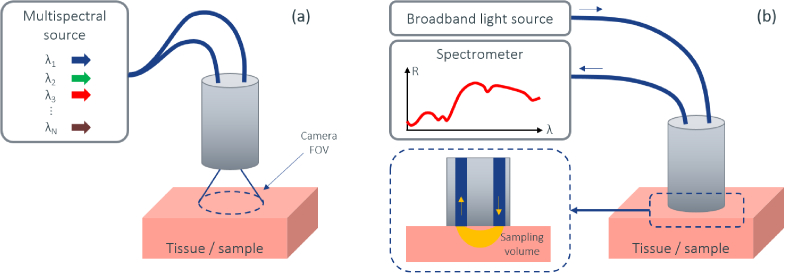
Schematic of sensing modalities of the system, with (a) wide-field non-contact diffuse imaging using multi-spectral or broadband illumination giving good spatial resolution and (b) DRS providing excellent spectral resolution. Diffuse imaging can either capture a colour image in a single frame using white-light illumination with an RGB image sensor, or stacking of sequentially acquired frames under monochromatic illumination for a multi-spectral approach. In contrast, DRS uses a spectrometer to measure broadband light that has diffusely propagated between the two fibres, thus probing a sampling volume with higher spectral resolution.

Broadly speaking, the image sensor captures diffusely reflected light from a wide field-of-view and spectral information can be gained either using multispectral illumination or Bayer spectral filters on the sensor. In contrast, DRS is a contact measurement and reflection data is collected from a sampling volume that typically penetrates a few mm into the tissue depending on wavelength and tissue type and inter fibre distance. In other words, where the image sensor records many spatial sampling points with a relatively coarse spectral resolution, the DRS system collects data from a 3-dimensional sampling volume with excellent spectral resolution. This is illustrated in Fig. 2. The two sensing modalities are complementary and a dual modality system has the potential to provide simultaneous high-resolution spectral and spatial information. The image sensor assists in the navigation and determining the region of interest, and the DRS can provide diagnostic information in this region. The camera-on-tip endoscope presented in this work is designed for dual operation with the image sensor and DRS in mind, as previously discussed.

At the core of the DRS system used in this work is a high-performance spectrometer (Wasatch Photonics, WP-VISNIR-R2-IC, 400-1080 nm, 1 nm resolution) with a temperature-controlled detector array for low-noise and stability. The illumination source is a fibre-coupled halogen (Thorlabs, SLS201L/M, 360 - 2600 nm) with −132 mireds colour temperature balancing filter (Thorlabs, FGT165M). The filter flattens the emission spectrum to ensure a significant SNR improvement across the measurement range. A fibre-only probe was designed to evaluate the DRS system separately to the The image sensor. However, it is important to notice that the same functionality can be achieved using the fibres in the camera-on-tip endoscope which can be put in contact with the tissue when required. Combining the two system requires an optical switch to collect diffusely reflected light with a spectrometer. The two systems were kept separate here for simplicity because the experiments were conducted remotely. The probe consists of a 400 μm 0.39 NA source fibre (Thorlabs, FT400EMT) and a 365 μm , 0.22 NA detector fibre (Thorlabs, FG356LEC). The cleaved fibres were positioned with a separation of 2.0 mm using a 3D printed housing. The system allows for a contact measurement to be made on tissue with data acquired in 80 ms. Calibration is performed using a spectralon target (SphereOptics, SG 3052). The next research step is the integration of both modalities, imaging and DRS, into a multimodal system using an optical switch. This system will be able to use the image data for guidance and navigation, with spatially resolved spectral data highlighting regions of interest. The system can be navigated to these regions and a DRS contact measurement can be conducted for verification.

### 2.3. Pseudo-colour visualisation of DRS for intuitive data presentation

In this section, we describe a novel approach to convert a full DRS spectrum to a single colour, which offers an intuitive way to interpret the complex DRS spectra to clinicians in an intuitive format that aligns with what is seen by the human eye and a image sensor. There is no unambiguous way to convert a spectrum to RGB space. However, the CIE 1931 colour matching functions x, y, z match the chromatic response of the cone cell in the human eye and offer one possible way to calculate the tri-stimulus values X, Y, Z of a spectrum S( λ ) similar to response of human eye [30]. These values can further be converted to RGB using standard functions in e.g. MATLAB for a given white point, such as D65 cold white illumination. Using these values the spectral data obtained by the DRS system is colour mapped to different shades of red, indicating higher or lower amounts of oxygenation in the tissue. In this way, we convert a full spectrum to a single pseudo-colour, which can be tracked over time. Whitenening of the colour corresponds to a change in oxygenation in the spectrum.

### 2.4. Study protocol

The porcine model was selected because of the anatomical and physiological similarities with the human heart and vascular system. Two adult animals were used in the study, which was conducted under approval of the protocol and ethics review board (Center for Cardiovascular Research and Development, American Heart of Poland Inc, TYN 2407-20). The animals were sedated with intramuscular ketamine (10 mg/kg) and xylazine (0.05 – 0.2 mg/kg) and anesthetised with inhaled isoflurane (1–3%). Heparin (150-200 IU/kg) was administered for systemic anticoagulation. The animals were placed in dorsal recumbency and electrocardiography was established.

After induction of anaesthesia, vascular access was obtained through the femoral artery. Guidewires and catheters were navigated using standard techniques and angiography, and the camera-on-tip endoscope was introduced through a large bore 9 Fr venous sheath. A balloon catheter was introduced into the left anterior descending artery (LAD), where inflation and deflation were used to control vessel occlusion and reperfusion to achieve hypoxia.

**Fig. 3.**
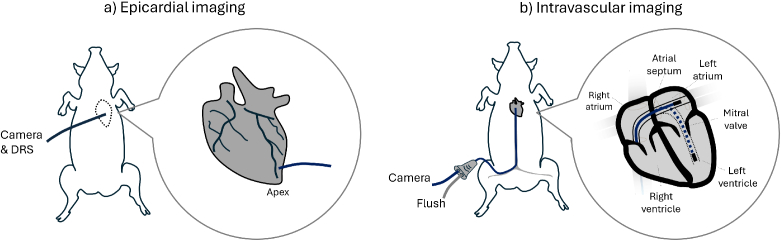
Schematic of study protocol. a) A left thoracotomy procedure was performed to image and perform DRS measurements of the epicardium. The probe was held near the apex. b) Using standard femoral artery catherisation, the endoscope was navigated to the right atrium and further through the septum to the left atrium and ventricle. The camera-on-tip endoscope was operated inside the guiding catheter with saline flush applied to clear the blood-filled FOV in front of the image sensor. A separate balloon catheter was inserted into the LAD to control vessel occlusion in both animal models.

An illustration of the study protocol is shown in Fig. 3. A left thoracotomy procedure was performed in the first animal to give access to the epicardium. Imaging the epicardium eliminates the challenge of a blood filled FOV and images were recorded of the area of the apex while inducing hypoxia. DRS data was recorded from the same area with a separate probe in contact with the tissue.

In the second animal, the 9 Fr venous sheath was inserted into the right atrium and images were acquired of the interatrial spectrum before performing a transseptal cross. The endoscope was navigated to the left atrium before passing through the mitral valve to the left ventricle. This follows the standard clinical workflow for a range of common cardiac procedures such as PVI. Images of the pericardium were recorded at relevant stages in the procedure. In particular, images were acquired continuously of a fixed FOV to observe the tissue colour change while inducing myocardial ischemia. Flushing was provided both through the larger guiding catheter using an automatic pump. As will be discussed in the following, holding the guiding catheter against pericardium provides an efficient way to clear the FOV from blood and provide tissue image data over a duration of minutes. The study was carried out in December 2020 during the COVID-19 pandemic. As a consequence, the procedure was performed by the surgical team in Poland with a video link to Ireland showing the operating theatre and fluoroscopy imaging. The imaging and DRS systems were designed for remote control and troubleshooting.

## 3. Results

### 3.1. Cardiovascular imaging for surgical guidance and diagnostics

To demonstrate the imaging performance and quality of the camera-on-tip endoscope, we successfully tested the system with extensive imaging of the epicardium and with real-time intracadiac of the cardiac walls. In particular, we successfully recorded images continuously over a duration minutes in the blood-filled cardiac cavities. Insertion of the camera-on-tip endoscope through the femoral artery and navigation to atria was performed with ease, and images and video were recorded throughout the procedure.

**Fig. 4.**
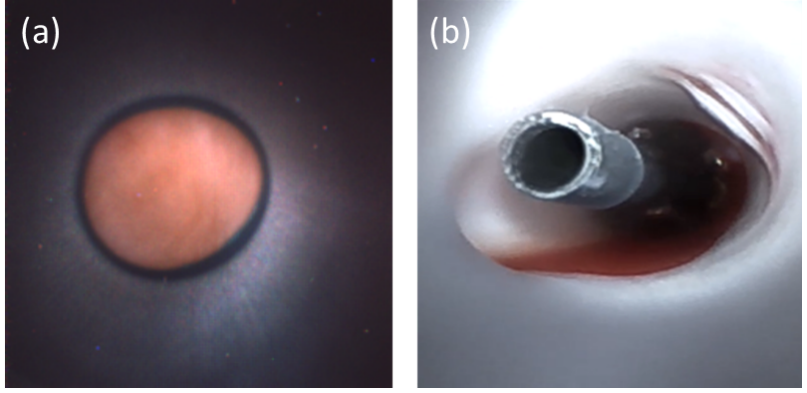
Two methods for clearing the field of view of the image sensor. (a) uses a catheter held against the heart wall with saline flush to clear the blood field. (b) uses a balloon catheter to clear the blood field. Note that the balloon catheter image only was captured in a porcine model in a different study.

The camera-on-tip endoscope cannot image through the blood field in a meaningful way. In order to clear the field of view two methods are demonstrated. In Fig. 4(a) a guiding catheter is placed in contact with the heart wall, and a saline flush clears the field of view, effectively isolating a region of tissue for imaging. The camera-on-tip endoscope can then be positioned spatially along the longitudinal direction of the guiding catheter to obtain live video of the isolated region with appropriate focus. In Fig. 4(b), a balloon catheter is inflated to isolate the region of interest from the blood field similarly through obstruction. The image sensor is then able to image the tip of the balloon catheter and other areas of interest. The camera-on-tip endoscope also has an integrated flush channel that allows for clearing the FOV immediately in front of the image sensor and removal of debris obstructing its FOV. In this study, the flushing was predominantly provided through the guiding catheter. With the guiding catheter positioned against the cardiac wall, this method gave a clear field-of-view over a duration of several minutes.

**Fig. 5.**
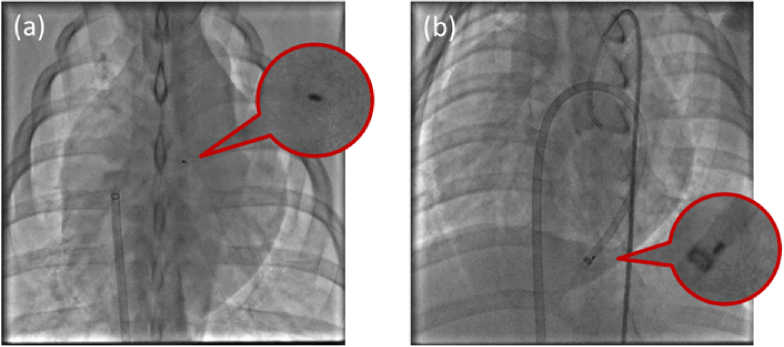
Fluoroscopy images of camera-on-tip endoscope. The endoscope is inserted through the right femoral artery and arrives in the right atrium through the inferior vena cava. (a) Transseptal puncture has been performed and the endoscope is now located in the left atrium. b) the endoscope has been bent, passed through the mitral valve and now located in the left ventricle and approximately at the apex.

The fluoroscopy images in Fig. 5(a) and (b) show fluoroscopic images mid procedure. The guiding catheter is clearly visible. In Fig. 5(a) the transeptal puncture has been performed. The system with marker band can be seen crossing from the right side of the heart to the left side. In Fig. 5(b) the endoscope has been bent further and has been navigated past the mitral valve and navigated to the apex of the heart.

**Fig. 6.**
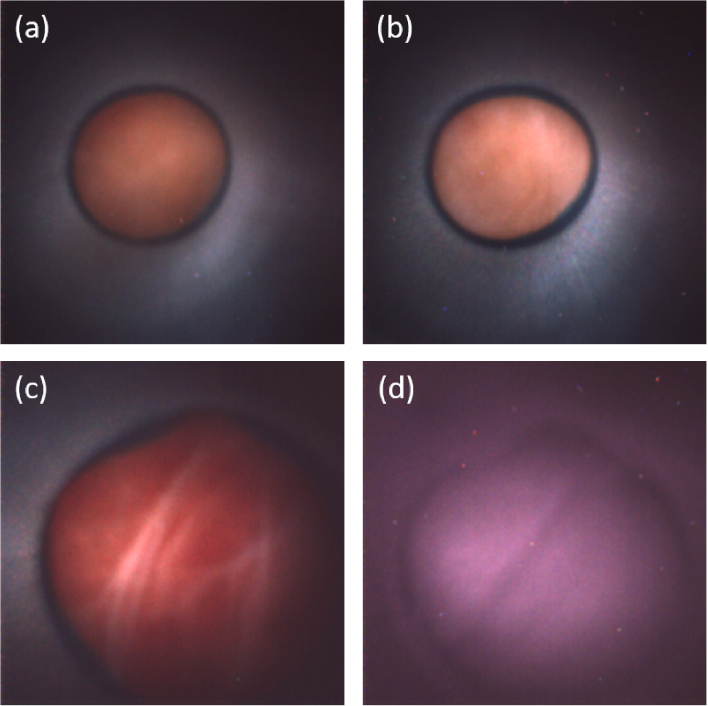
Examples of images acquired *in vivo* in the second porcine model in different chambers of the heart. The camera-on-tip endoscope was first inserted into the right atrium where images were captured of the septum in (a)-(b). After performing a transseptal puncture, images were recorded of the left ventricle under (c) visible and (d) near-infrared illumination. A video recording is available online (see Visualization 1, "Video1.mp4", 8.38 MB) showing continuous imaging of the cardiac walls of the beating heart over 2 minutes with white-light laser illumination. The image in (c) is a still from the video.

In Fig. 6(a) and (b) images of the septum were acquired under reconstructed white-light laser illumination. Note that the endoscope can be maneuvered closer to the tissue filling a greater field of view if desired. This can be seen in Fig. 6(c) and (d). In these images the left ventricle is imaged. The system allows for flexibility in imaging modalities and Fig. 6(d) shows the imaging under NIR illumination. The NIR light has a greater penetration depth in tissue and allows for imaging at some depth. Figure 6(c) and (d) are imaging the same tissue region and the large diagonal feature is visible in both images. Video data of the procedure is included in the supplementary online material. In the video data there is an occasional blood flow incursion into the guiding catheter. This can be quickly cleared with saline flush through the guiding catheter.

### 3.2. DRS measurements of ischemia and reperfusion

**Fig. 7.**
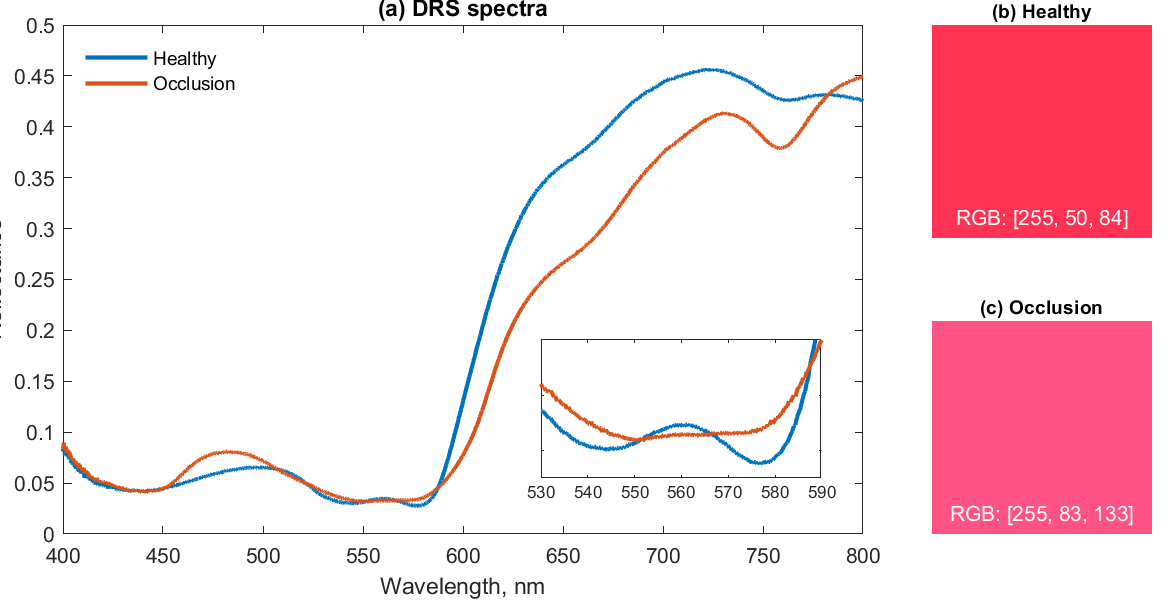
(a) DRS spectra of the epicardium of the first animal model. The two spectra are for healthy oxygenated tissue and with occlusion of the LAD, respectively. The spectra are averaged over 3 seconds to remove artefacts from the heart beat. (b)-(c) The DRS spectra for healthy and occluded tissue are each converted to a single RGB colour with CIE 1931 colour matching functions to match the response of the cone cells in the human eye, which suggests a whitening of the tissue during the occlusion.

We recorded DRS measurements of the epicardium continuously through several cycles of ischemia and reperfusion to demonstrate that the tissue state can be detected with our system. Representative spectra of healthy tissue and during the occlusion are shown in Fig. 7(a). The characteristic double absorption peaks from oxyhemoglobin at 540 and 575 nm are clearly visible in the healthy DRS spectrum as a decrease in reflectance. These features disappear during the occlusion, where the spectrum is dominated by the deoxyhemoglobin absorption peak around 555 nm. The spectra further show an increased reflectance in NIR from deoxyhemoglobin (decrease in absorption). This is in agreement with what is reported in the literature [31].

A DRS spectrum is rich with information and can be hard to interpret without an established reference. Here we propose a spectrum-to-colour conversion that can potentially give an intuitive interpretation of the tissue state. Figure 7(b)-(c) show the spectrum-to-colour conversion described previously, where the full spectrum is converted to an RGB colour using the CIE 1931 colour matching functions that match the response of the cone cells in the human eye. As can be seen in the figure, the RGB colours calculated from the spectra change to a paler shade of red during the occlusion as expected from the decreased reflectance. It should be noted that the exact colour scale depends on the CIE XYZ to RGB conversion, which has not been calibrated here. This spectrum-to-colour conversion offers a potential novel and intuitive way to interpret the DRS measurements that can be merged with the camera data. Additionally, it is important to note that any colour map that portrays clinical significant difference to a clinician could be used here, and the goal is to visualize only the change. Hence, for example, with sufficient calibration ischemic tissue could be labelled "red" and healthy tissue "green", with the intermediate values appearing in "amber", in a traffic light approach. In the all-red colour map chosen above, the calculated colour change is strongly exaggerated but nonetheless gives clear feedback to the clinician that a change has occurred. Reflectance measurements sample a volume of tissue that, depending on the wavelength and tissue composition, can penetrate up to a few mm into the tissue. The spectral colour can therefore not be expected to be what a human eye or image sensor would see.

**Fig. 8.**
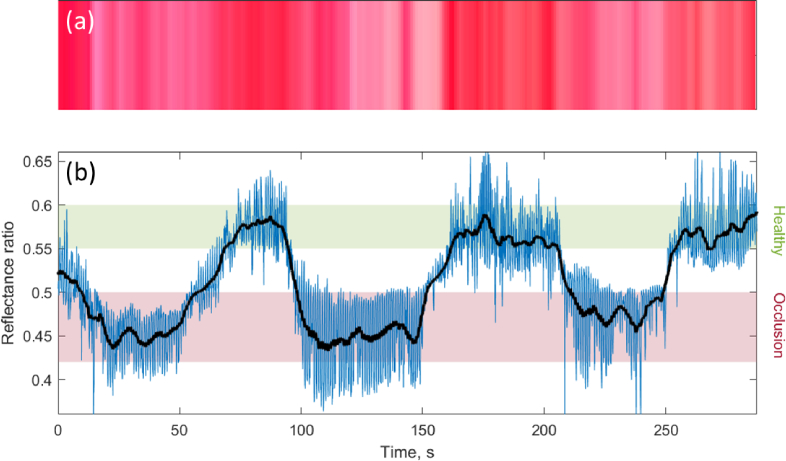
Time series of DRS reflectance ratios and corresponding spectrally converted RGB values. The reflectance ratio is calculated as the deoxyhemoglobin absorption peak at 555 nm divided by the sum the oxyhemoglobin absorption peaks at 540 and 575 nm, respectively. The ratio-metric regions coarsely corresponding to healthy and occlusion are indicated in (b) and the black line is a moving average low-pass filter to remove the heartbeat. The RGB values are calculated from 1-second averaged spectra to remove artefacts from the heartbeat.

In Fig. 8 we show a time series of the spectrum-to-colour conversion together with a reflectance ratio, calculated as R(555nm)/(R(540nm)+R(575nm)) , where R is the average reflectance in a 5 nm band. These spectral bands are the deoxyhemoglobin absorption peak at 555 nm and the sum the oxyhemoglobin absorption peaks at 540 and 575 nm, respectively. We observe a clear change in the average DRS reflectance ratio over the course of the measurement which spans multiple occlusion cycles, where healthy tissue shows a higher reflectance ratio than when the tissue is occluded. We also observe that the tissue recovers relatively quickly once the occlusion is lifted, within 10-20 seconds. There appears to be no long lasting effects due to the multiple occlusions with the levels returning to the same range each time. These results show the sensitivity and fast response of DRS and its applicability in the *in vivo* cardiovascular space. Similar results to those captured on the epicardium are expected for DRS measurements of the endocardium.

### 3.3. Cardiac imaging plethysmography

The amount of light back-scattered from tissue that is captured by the imaging sensor is related to changes from perfusion, heart rate and respiration. Here we show that physiologically relevant data can be calculated by careful analysis of these intensity variations. However, the field of view can be changing rapidly with motion. By using the guiding catheter to exclude the blood field, the endoscope is stabilised and a region of interest (ROI) can be collected in the centre of the image that is not obscured by the guiding catheter. To this end, we have developed an algorithm to capture only this central region, which works as follows: firstly, we select the red channel as it has a good contrast between the tissue and guiding catheter. This layer is then binarised using a standard adaptive algorithm with a sensitivity of 0.6. To enhance the contrast of the circular ROI of the guiding catheter, the binary image is convoluted (closed) with a Gaussian blur using a disk with 3-pixel diameter. The resulting binary image clearly shows the circular diameter of the guiding catheter, which is fitted with a standard circle-fitting algorithm. In some cases, multiple circles are found but the fit with the best fitting parameter is chosen. When the tissue is not visible due to incursion of blood, the walls of the guiding catheter are also not visible. Frames with poor circle fit are in this way disregarded. When imaging outside of the guiding catheter a central region of interest without fitting can be used.

**Fig. 9.**
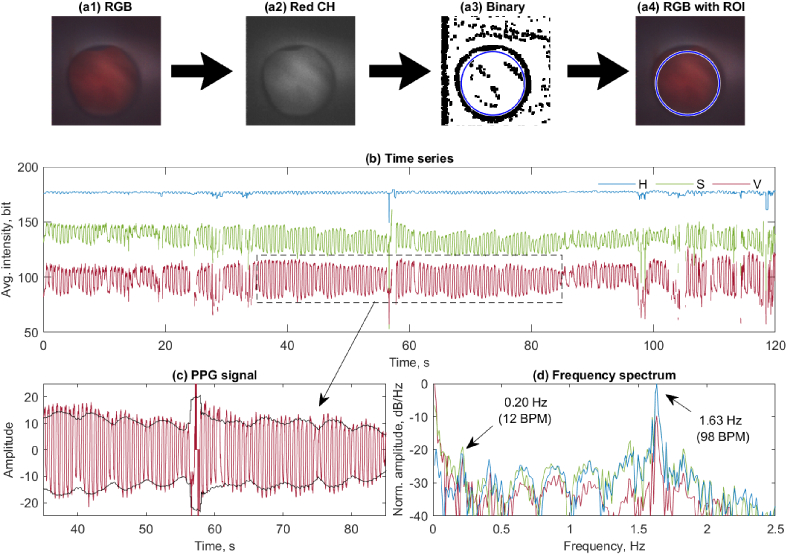
Data analysis of video captured inside of the left ventricle. Images (a1)-(a4) show the circle fitting algorithm which detects the relevant tissue pixels, using the outer guiding catheter as a reference. The red channel (a2) is used and the image is binarised. The largest circle is detected in the binary image (a3), and this results in the circular ROI (a4). This process is repeated for each frame. Frames where no circle is detected are discarded. Using this data a time series of each of the HSV channels is plotted (b) showing the variations in average intensity of the region in each frame. A section of the time series is selected for analysis (c) with the average subtracted and and envelope function added to show the respiration rate. The normalised Fourier transform of each channel over this time segment revels features in frequency space (d). This shows a sharp peak at the heart-rate of the animal at 98 bpm and another peak at 12 bmp, the respiratory rate of the animal.

In Fig. 9(a1)-(a4) an extracted frame is shown, from which the red channel is extracted. The previously described binarisation and ROI extraction algorithm extracts the circular region of interest. The average H, S and V values are extracted from a 2-minute video sequence of the left ventricle. The ROI used for the calculations is chosen as a circle with 90% of the radius found in the fit to remove any boundary effects such as blood leaking into the guiding catheter. The calculated values are shown as a time series in Fig. 9(b). This Photoplethysmogram (PPG) data is shown in Fig. 9(c). From such a time series standard physiological data, such as heart rate and respiration, can be clearly observed. The respiration is more clearly seen in the normalised data. Figure 9(d) shows a fourier transform of the signal with peaks at both the respiratory rate of the porcine model at 12 beats per minute (BPM) and a heart rate of 98 BPM. It is highly encouraging that the values can be clearly observed over a 2 min window. It is also the first demonstration of *in vivo* and intra-vascular PPG data collection using a image sensor. With further development, this technique can potentially be expanded from a single averaged PPG signal to imaging PPG with spatial perfusion information.

### 3.4. Tissue oxygenation from image data

**Fig. 10.**
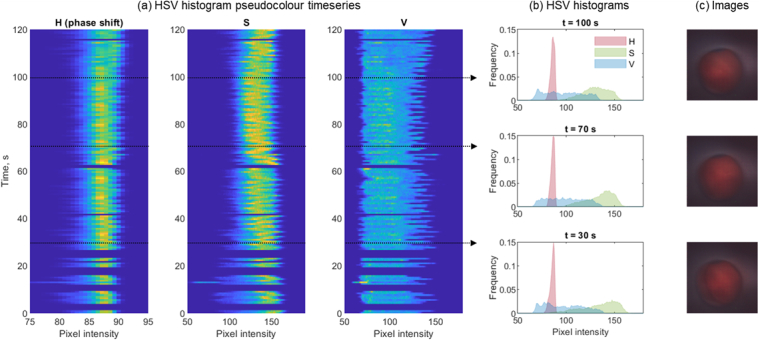
(a) Histogram pseudocolour timeseries of the HSV layers (Hue, Saturation, Value) from the same video used in Fig. 9. The data is averaged over 20 s to remove motion artefacts and the data is disregarded where no circle could be fitted as described in Fig. 9. (b) Histograms shown at selected times to more clearly highlight the changes in the S and V channels. (c) The corresponding images averaged over the same 20 s duration as the histograms in (a)-(b).

To explore if we can differentiate ischemic tissue from healthy tissue using the micro image sensor, we examine the distribution of pixel intensities in each of the colour channels of the imager following arterial occlusion. In order to approximate the human visual response, images are converted to the HSV colour space. Figure 10(a) shows the aggregated pseudocolour histograms of the same imaging data used in Fig. 9, averaged over 20 frames per histogram. Ischemia is induced at around 30 s in the time series. Figure 10(b) shows histograms at selected times during the occlusion cycle. It is observed that there is a shift in the distribution over the duration of the induced ischemia, most apparent in the saturation value. This can be interpreted as a change in absorption between oxy and deoxy hemoglobin, which is in agreement with the DRS measurements presented above. The images in Fig. 10(c) show the time-averaged images used in the calculation of (b). The subtle colour changes are more clearly observed in the histograms than the images, which will be explored further in future work.

## 4. Discussion

The micro imaging sensor’s small footprint allows integration into miniature endoscopes as presented in this work, as well as enabling other small footprint tools to be sensorised with a camera. The main output of the micro sensor is an image, either monochrome or in colour, which is used in navigation and guidance at location *in vivo*. In cardiovascular imaging, the micro image sensors’ footprint allows it to be integrated into 3-8 Fr (1-2.5 mm diameter) endoscope devices, which can be navigated to small blood vessels through a slightly larger guiding catheter. The small footprint of the image sensor and successful integration into a 2.30 mm endoscope system can be seen in Fig. 1. The sensor is usually integrating in a forward facing modality but could be arranged to be side-facing. The returned image then provides a clinician with vision of an area of interest.

Applications of such a guidance image can be divided into two categories, image enhanced guidance and diagnostics. We define image enhanced guidance as navigation to a feature using an *in situ* image, and diagnostics as the ability to visualise a feature of interest *in vivo*, for example a lesion or device, and the ability to gather additional data using a DRS sensor, for example. Imaging involves recording an image of an area, which is usually difficult or impossible to visualise. The camera-on-tip endoscope allows for live vision on location. This includes the verification of correct device placement in cardiovascular procedures. More in the diagnostic direction the image inside of the vessel can aid diagnostic tools by locating of areas of interest for further analysis such as with a contact measurement DRS probe as is presented in this work. Or the image itself can be used to obtain diagnostic information by looking at the colour change in the pixels, extracting information about colour changes which may be due to tissue oxygenation, and either correlating them to a clinically significant marker or extracting information such as heart and breathing rate from such colour changes. We note that co-registration of the wide-field imaging and contact-based DRS must be considered to successfully merge the two sensing modalities. The guiding catheter provides a well-defined FOV where multiple DRS measurements can be performed as needed.

Various procedures in interventional cardiology involve placing devices, such as pacemaker leads, stents, and valves, under fluoroscopic guidance. During minimally invasive valve repair interventions, such as Transcatheter Aortic Valve Implantation (TAVI), a new valve is delivered through the blood vessels to location in the heart for example to treat aortic stenosis [32–34]. While stent placement has been verified using OCT and IVUS technology, often the surgeon lacks vision inside the vasculature and relies on repeated fluoroscopic imaging. A front mounted image sensor can give directional information about where the endoscope is pointing to. During a transeptal cross procedure locating the correct puncture spot is critical; when navigating to a particular region in the heart the angle of this puncture spot may want to be adjusted. Image guidance and sensing *in situ* has the potential to reduce the reliance on fluoroscopy during this procedure. During a Pulmonary Vein Isolation procedure (PVI) tissue is ablated in order to treat arrhythmia. Electrodes may not capture incomplete or partial ablation. In these cases an image sensor and DRS system in combination can be used to validate transmural ablation and have the potential to reduce radiation exposure for doctors and patients and to speed up the procedure. The technology may also see use in recording procedures and verifying the efficacy and success of an intervention. A possible application of intra-catheter sensing technology may be in imaging the infected heart: Endocarditis is a clinical problem that progresses rapidly and modern imaging techniques have been limited in their ability to diagnose, especially early [35]. It can occur as a complication after device placement, and affects 0.5%-3% of patients within one year of the TAVI procedure alone [33]. The ability to guide navigation and deploy sensing tools inside of the heart may prove useful in verifying infection, and locating it, when non-invasive technologies fail to be conclusive. For any clinical use case, it is important the camera-on-tip endoscope fits with the standard workflow and provides reliable and clinically beneficial data that is easy to interpret.

Recent work has investigated the many advantages of micro CMOS imagers for cardiac applications [21–24], but these studies have generally focused on visualisation only with no quantitative information derived from the imaging sensor. Here we have demonstrated that physiological parameters can be calculated from the imaging sensor, which we expect will provide additional value to clinicians. For example, it has been demonstrated that imaging PPG can provide information on the perfusion status of tissues during surgery [36]. In this work, we used the full FOV to calculate a PPG signal, but this can potentially be extended to generate PPG perfusion maps from a micro CMOS sensor with improved image analytics. Similarly, we are expecting that multi-spectral imaging will provide additional quantitative insights such as spatial mapping of the tissue oxygenation state and can further significantly enhance the image contrast to highlight, e.g., vasculature. This can potentially be used to evaluate the success of cardiac ablation procedures.

Operation of an image sensor in a vessel comes with the additional challenge of clearing the blood field. Two methods are generally adopted for clearing the FOV. The first involves inflation of a balloon catheter within the blood vessel clearing the FOV and allowing imaging of the walls of the vessel. This is not always an optimal solution, for example in the heart where the obstruction of an area with a balloon is not possible or could be detrimental to the patient. The second method involves the guiding catheter being used to shield the sensor from continuous blood flow. A saline flush can then be used to clear the FOV making imaging possible. We distinguish between two methods of flushing, the macro-flush where the guiding catheter is used as the flush channel, and the micro-flush where the flush channel is integrated into the camera-on-tip endoscope.

Specular reflections are direct reflections that occur on glossy uneven surfaces of biological tissue and can cause problematic image artefacts that detract from the image quality on an already resolution limited sensor by creating bright spots where the underlying features are obscured. Movement of the sensor during a procedure can help identify these reflections since they will move, however removal, reduction and prevention is preferred. We have previously demonstrated two methods of specular reflection removal using micro image sensors, using an algorithmic approach and a static cross-polarisation approach [37]. In a cardiovascular application due to frequent movement within the body a static polarization approach would be preferred and may be implemented in a future design. All aspects, from illumination wavelength and delivery, to mechanical integration, and lensing at the tip of the device, will be considered and improved further in future work.

For general imaging, the coherent nature and resulting speckle pattern have been found to interfere with image quality in this application space, even though the speckle seems to be reduced due to Brownian motion in the liquid field. As a result an LED-based source is currently preferred for illumination, however future work is looking at using a de-speckling system in the laser-based illumination pipeline [38].

The output image lends itself to various image-processing techniques, many of which can be implemented in real or near real-time. The image sensor operates at around 40 frames per second (FPS), and the low resolution allows for rapid processing. The addition of a field-programmable gate array (FPGA) scan help to further optimize image processing algorithms in a clinical systems [39].

## 5. Conclusion

In conclusion, we present a 7 Fr (Ø2.3 mm) camera-on-tip endoscope system for cardiovascular guidance and diagnostics applications. The system uses fibre-coupled multi-spectral illumination methods to image in a blood-filled field of view. We demonstrate that the endoscope can be navigated to challenging cardiac regions through the femoral artery in a porcine model. Specifically, we present images recorded *in vivo* of the inter-atrial septum, and cardiac walls in the left atrium and left ventricle. To the best of our knowledge, this is the first time a camera-on-tip endoscope has been navigated through the femoral artery to the left atrium and ventricle with images recorded *in situ*. We further show that physiologically relevant parameters such as heart rate and respiration can be extracted from the imaging data. A method is outlined for measuring the tissue oxygenation state from the image data and merging with DRS data. The results presented in this work show the potential for cardiovascular imaging with camera-on-tip endoscopes to provide physiologically relevant data and device placement that can improve the clinical outcome.

## Supplemental information

Visualization 1 Camera-on-tip angioscope video recording of cardiac wall of porcine model https://doi.org/10.6084/m9.figshare.27101140

## Data Availability

The data and analysis code that support the findings of this study are openly available at [40].
